# Unleashing the Blood Clots: Revolutionizing Acute Pulmonary Thromboembolism Management With Endovascular Techniques

**DOI:** 10.7759/cureus.77419

**Published:** 2025-01-14

**Authors:** Arindam Pande, Arnab Bera, Arpan Chaudhuri, Somnath Dey

**Affiliations:** 1 Department of Cardiology, Medica Superspecialty Hospital, Kolkata, IND; 2 Department of Chest Medicine, Medica Superspecialty Hospital, Kolkata, IND; 3 Department of Internal Medicine, ILS Hospitals, Kolkata, IND; 4 Department of Cardiac Anesthesia, Medica Superspecialty Hospital, Kolkata, IND

**Keywords:** deep vein thrombosis (dvt), inferior vena cava filter, pulmonary embolism, thrombectomy, thrombosis

## Abstract

Venous thromboembolism (VTE), encompassing deep vein thrombosis (DVT) and pulmonary embolism (PE), are the leading causes of mortality and morbidity worldwide. Embolization of deep vein thrombi into the pulmonary arteries is thought to be the most common cause of PE. While the standard management of acute PE involves anticoagulation, mechanical thrombectomy, and, in certain cases, the use of inferior vena cava (IVC) filters, clinical scenarios with contraindications to anticoagulation necessitate alternative therapeutic approaches. We present the case of a 77-year-old female who presented with acute PE and multiple comorbidities, including active hematuria. The patient was successfully managed by mechanical thrombectomy using the Penumbra Indigo^®^ Aspiration System (Penumbra Inc., Alameda, CA) and subsequently underwent temporary implantation of an IVC filter to prevent the risk of recurrence. This case contributes to advancing current knowledge in the management of acute PE, particularly in frail, elderly patients with multiple comorbidities, and reinforces the critical role of personalized, multi-modal therapeutic approaches in improving survival rates.

## Introduction

Venous thromboembolism (VTE) encompasses pulmonary embolism (PE) and deep vein thrombosis (DVT). Among the symptomatic VTE patients, nearly two-thirds have DVT, whereas about one-third of such patients have PE [1,2]. The universal prevalence of VTE ranges from 0.75 to 2.69 cases per 1,000 population and two to seven cases per 1,000 in the elderly [3]. Indian data suggest that the overall incidence of PE in adults is 15.9% and that PE is a cause of mortality in 80% of such patients [1,4]. PE is the most severe complication of DVT, and it occurs when embolic venous thrombi are caught within the branching vasculature of the lungs. These thrombi frequently develop within pelvic veins or in the veins of the leg, and around half of all deep-vein thrombi embolize the lungs [5]. The manifestation spectrum of PE ranges from clinically silent form where the patient may be completely asymptomatic, to massive PE where the patient may present with hemodynamic collapse requiring ionotropic support, and sometimes fatal events [6]. Hence, prompt diagnosis and suitable treatment of this condition can dramatically decrease its attendant mortality and morbidity [7]. In this report, we present the case of a 77-year-old hypertensive female who was diagnosed with acute pulmonary thromboembolism and was managed successfully by mechanical thrombectomy and an inferior vena cava (IVC) filter implantation.

## Case presentation

**Figure 1:**
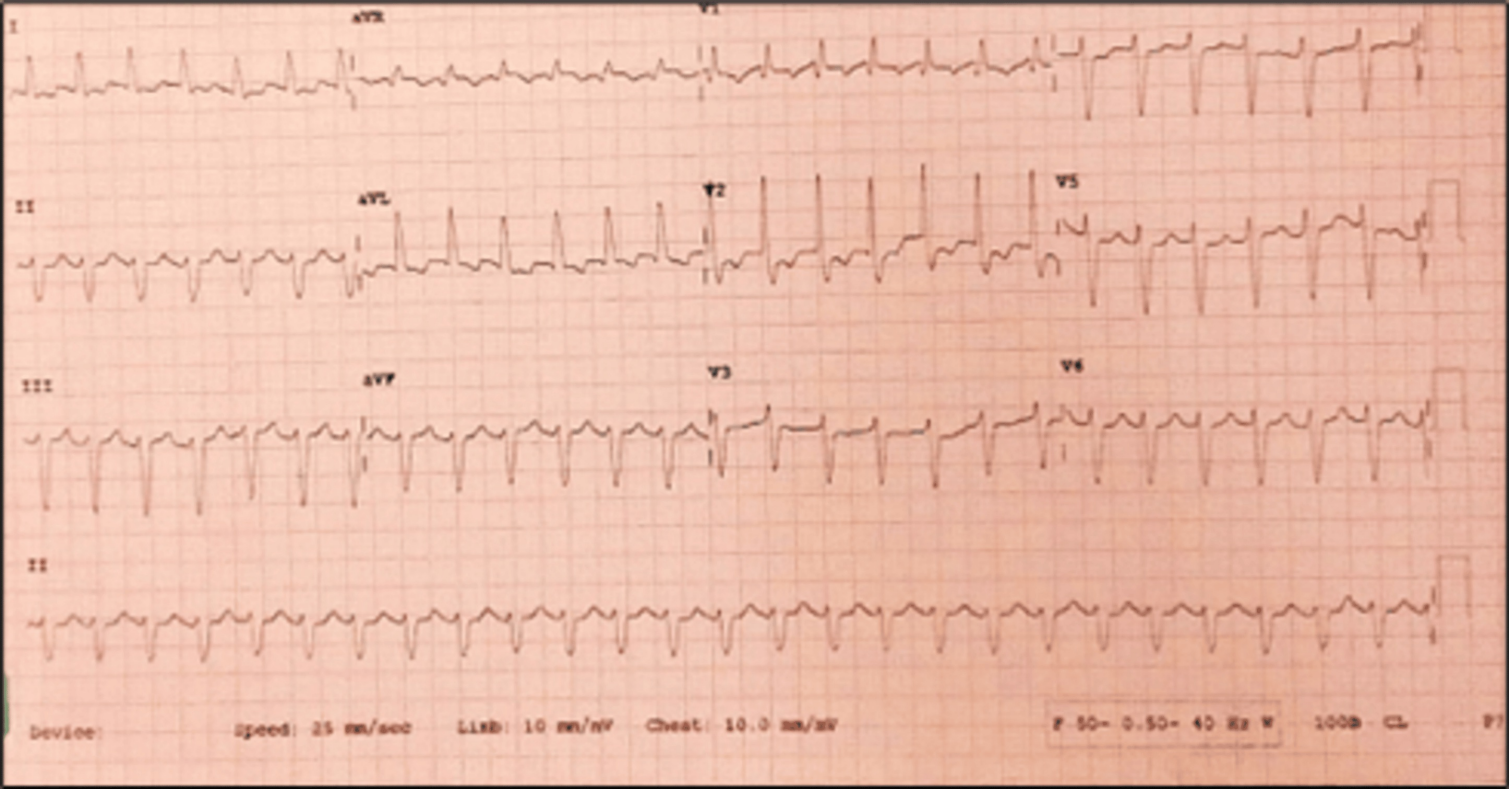
Electrocardiogram showing atrial fibrillation with a high ventricular rate and a right bundle branch block with left axis deviation

**Figure 2:**
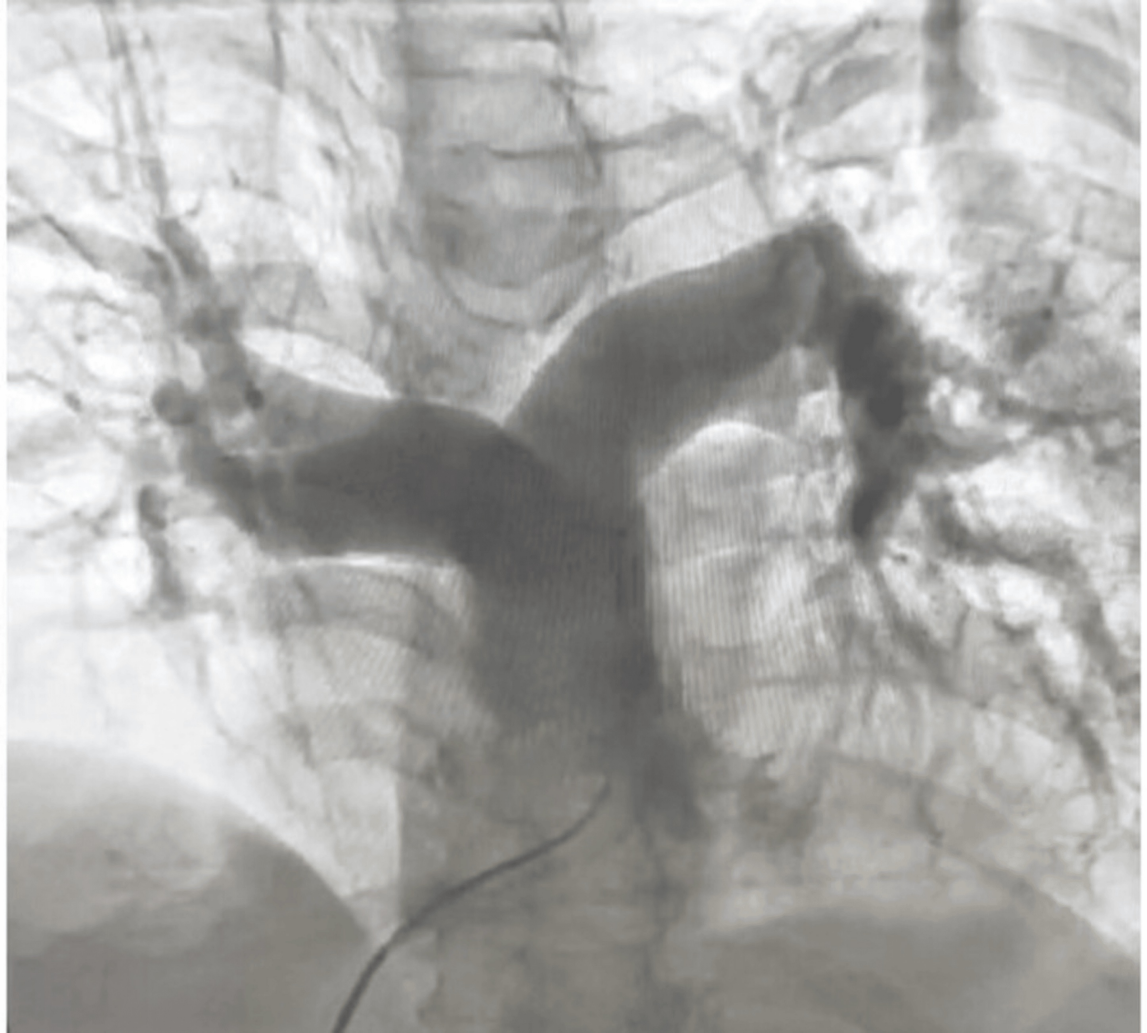
Contrast-enhanced pulmonary angiogram showing thrombus as multiple filling defects in both pulmonary arteries

A 77-year-old hypertensive, non-diabetic, euthyroid frail female presented with severe shortness of breath, hematemesis, and hematuria. Clinical investigations revealed urinary tract infection and acute kidney injury. The patient had a history of previous hospitalization with a right-sided Colle’s fracture and heart failure with preserved ejection fraction. On admission, the patient’s body temperature was 96.1°F, pulse rate was 125/minute, blood pressure was 110/70 mm Hg with ionotropic support of noradrenaline infusion (8 mcg/hour), and oxygen saturation (SPO_2_) was 88%-95%. Table 1 summarizes the baseline blood work results. Electrocardiogram showed atrial fibrillation with high ventricular rate, right bundle branch block, and left axis deviation (Figure 1). Pulmonary angiography demonstrated multiple filling defects in both pulmonary arteries (Figure 2).

**Table 1:** Blood parameters at baseline

Variables	Values at baseline
Hemoglobin	8.8 g/dL
White blood cells	15,600 cells/μL
C-Reactive protein	124 mg/dL
Serum urea	76 mg/dL
Serum creatinine	1.7 mg/dL
N-terminal pro-brain natriuretic peptide	3,244 pg/mL
High-sensitivity troponin T	19 pg/mL
Serum sodium	132 mEq/L
Serum potassium	3.2 mEq/L

**Figure 3:**
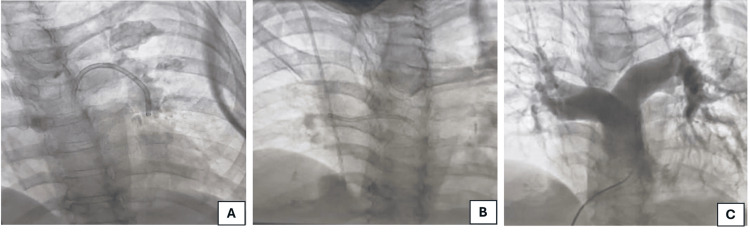
Angiogram showing (A) thrombus aspiration in the left pulmonary artery; (B) thrombus aspiration in the right pulmonary artery; and (C) final result after thrombus aspiration

**Figure 4:**
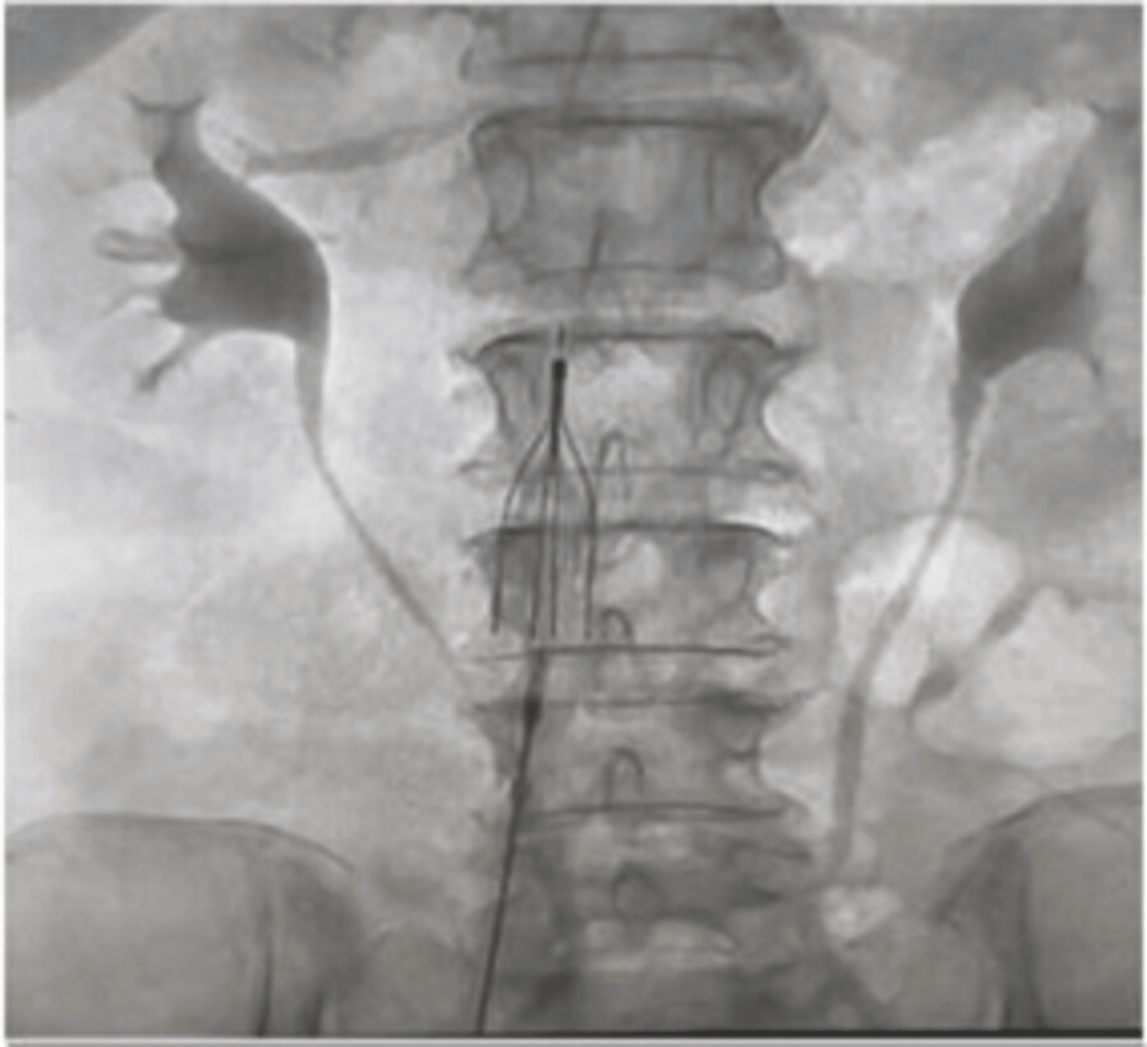
Angiogram showing implantation of a transient inferior vena cava filter at the suprarenal inferior vena cava through the right femoral vein

Echocardiography revealed dilated right atrium, right ventricle, and IVC with inspiratory collapse <50%. The patient was diagnosed with acute pulmonary thromboembolism and urinary tract infection with active hematuria and anemia. She was shifted to the critical care unit (CCU), a Foley catheter was inserted, and intravenous antibiotics were initiated after collecting urine samples for culture and sensitivity tests. For pulmonary thromboembolism, anticoagulant medicines could not be initiated in the presence of active hematuria. Therefore, catheter-guided pulmonary thrombectomy and IVC filter implantation were planned under local anesthesia. Thrombus aspiration was done using Penumbra Indigo^®^ Aspiration System (Penumbra Inc., Alameda, CA). Figure 3 illustrates thrombus aspiration in the left pulmonary artery (Figure 3A), thrombus aspiration in the right pulmonary artery (Figure 3B), and the result after thrombus aspiration procedure (Figure 3C). After successful thrombus aspiration, an IVC filter was implanted through the right femoral venous access to prevent recurrent DVT (Figure 4). Both procedures were performed without any complications in the same sitting.

**Figure 5:**
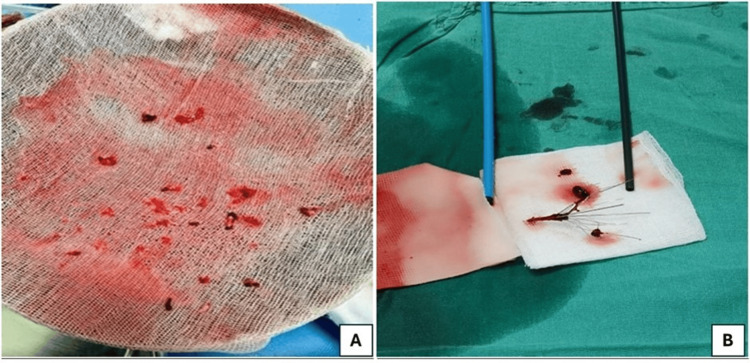
(A) Extracted blood clots from both the pulmonary artery; (B) Extracted inferior vena cava filter, which is filled with blood clots

After the procedure, inotropic support was gradually tapered off within 24 hours, and two units of packed red blood cells (pRBCs) were transfused as the hemoglobin dropped to 6.8 g/dL. Intravenous antibiotics were adjusted as per the culture sensitivity report. After four days of intervention, hematuria stopped. Therefore, oral anticoagulant apixaban was initiated. There was no subsequent recurrence of hematuria thereafter. The kidney function of the patient was improved, and she returned to sinus rhythm subsequently. On the eighth day of the intervention, the patient was shifted from CCU to the ward. After five days in the ward, the Foley catheter and central lines were removed, and the patient was discharged under home care supervision. After 26 days, the patient was re-admitted for IVC filter extraction. After admission, ultrasound-guided right jugular vein access was created to prevent bleeding complications as the patient was on oral anticoagulation therapy. Gunther Tulip^®^ vena cava filter retrieval set (Cook Medical LCC, Bloomington, IN) was used to retrieve the IVC filter. The IVC filter was captured with the snare, and the whole system was extracted through jugular access without complications. The extracted blood clots from both pulmonary arteries are depicted in Figure 5A. The extracted IVC filter, which was filled with blood clots, is represented in Figure 5B. Post retrieval of the IVC filter, the patient was discharged on the same day under stable conditions.

## Discussion

Acute PE is a clinical and pathophysiological syndrome characterized by obstruction of the pulmonary circulation due to endogenous or exogenous emboli that block the main pulmonary artery or its branches [8,9]. It is a critical and severe disease associated with high mortality. The major risk factors for acute PE are chronic cardiopulmonary disease, thrombosis of the lower limb or pelvic veins, long-term bed rest or inactivity, surgery, malignant tumors, pregnancy, trauma, oral contraceptives use, congenital prothrombotic conditions, etc. [8]. In addition, social disparities are increasingly recognized as important factors in understanding and improving cardiovascular and all other health outcomes. These barriers include a lack of education, lower income, higher rates of unemployment, frailty, and poor living conditions [10]. Our index elderly frail patient used to live alone, which might have played some role in the overall complicated clinical presentation. She had a history of previous hospitalization due to orthopedic issues, which is the predominant underlying risk factor for the development of DVT and PE.

Contemporary treatments for PE fall into four broad categories (i.e., systemic anticoagulation, systemic thrombolysis, catheter-directed thrombolysis, and mechanical thrombectomy) [11]. Anticoagulation is applicable for all forms of PE, while thrombolysis and other treatments are reserved for massive PE with hemodynamic compromise. In our case, the presence of active bleeding in the form of significant hematuria prevented us from using thrombolytic and anticoagulation therapy. Mechanical thrombectomy offers the advantage of quick clot removal while avoiding the bleeding risk complications linked with tSocial disparitieshrombolytic therapy. The choice of treatment depends on the individual patient's clinical presentation, hemodynamic stability, and risk factors [12]. In general, mechanical thrombectomy has several advantages over catheter-directed thrombolysis and systemic thrombolysis in the treatment of submassive and potentially massive PE [11]. The only randomized controlled trial comparing large-bore mechanical thrombectomy or catheter-directed thrombolysis in intermediate-risk PE published recently demonstrated that the large-bore mechanical thrombectomy had lower rates of clinical deterioration and/or bailout and postprocedural intensive care unit utilization compared with catheter-directed thrombolysis, with no difference in mortality or bleeding [13].

The Indigo Aspiration System (3.4-12 F) by Penumbra, Inc. (Alameda, CA) is a mechanical thrombectomy system designed for thrombus aspiration, comprising a catheter, separator, and vacuum pump. The Penumbra ENGINE facilitates thrombus removal by providing constant suction. The Indigo Separator^TM^ aids in catheter cleaning and thrombus fragmentation. Additionally, the Lightning^TM^ Intelligent Aspiration Tubing monitors blood flow in real time, through its dual pressure sensors. This enables the system to automatically regulate the level of aspiration, minimizing blood loss by providing intermittent aspiration around the venous section while maintaining constant aspiration near the clot [14]. The EXTRACT-PE trial (Evaluating the Safety and Efficacy of the Indigo Aspiration System in Acute Submassive Pulmonary Embolism) prospectively assessed the safety and efficacy of the Indigo Aspiration System in 119 patients with submassive acute PE. The results demonstrated that the Indigo Aspiration System significantly reduces the right ventricular-to-left ventricular ratio and major adverse event rate in this group of patients [15,16].

The IVC filters are indicated in patients with acute PE who have contraindications to anticoagulation (class I recommendation) and in patients with acute PE who have poor cardiopulmonary reserve and who are deemed to be at high risk of death if they develop another PE (class IIb recommendation, level of evidence C) [17]. Most patients with high-risk submassive and massive PE fall under the latter category. Regardless of the presence of concurrent lower extremity DVT, these patients should be considered for the insertion of a temporary IVC filter. The use of the IVC filter was associated with a reduced 90-day death rate in the International Cooperative Pulmonary Embolism Registry (ICOPER), with a hazard ratio of 0.12 (95% CI: 0.02-0.85). Furthermore, in a recent analysis by Stein et al., unstable patients with acute PE who received an IVC filter had a reduced in-hospital death rate than those who did not receive an IVC filter. Patients who receive removable IVC filters should be re-evaluated periodically for retrieval of the filter as soon as considered safe [7]. Hence, the present case underscores the efficacy of mechanical thrombectomy and implantation and timely removal of IVC filter in managing acute pulmonary thromboembolism, particularly in high-risk patients with multiple comorbidities.

## Conclusions

Multiple comorbidities of our patient with acute PE compelled us to formulate a tailored therapeutic strategy and meticulously chosen interventions. The utilization of advanced mechanical thrombectomy systems, such as the Penumbra Indigo^®^ Aspiration System, demonstrated promising outcomes in our critically ill patients. Additionally, the implantation of an IVC filter in a high-risk patient contributes to reducing the risk of recurrent thromboembolic events. Moreover, successful IVC filter retrieval resulted in a reduction of long-term risks associated with permanent in-situ devices. Large-scale randomized studies incorporating modern intervention strategies in the future will help formulate more clear guidelines in this field. Overall, the present case highlights the importance of prompt diagnosis, timely intervention, and comprehensive management plans as fundamentals in improving outcomes for patients with acute pulmonary thromboembolism.
